# Flowering time control in European winter wheat

**DOI:** 10.3389/fpls.2014.00537

**Published:** 2014-10-09

**Authors:** Simon M. Langer, C. Friedrich H. Longin, Tobias Würschum

**Affiliations:** State Plant Breeding Institute, University of HohenheimStuttgart, Germany

**Keywords:** wheat, flowering time, photoperiod, Ppd, copy number variation, epistasis, candidate genes, association mapping

## Abstract

Flowering time is an important trait in wheat breeding as it affects adaptation and yield potential. The aim of this study was to investigate the genetic architecture of flowering time in European winter bread wheat cultivars. To this end a population of 410 winter wheat varieties was evaluated in multi-location field trials and genotyped by a genotyping-by-sequencing approach and candidate gene markers. Our analyses revealed that the photoperiod regulator *Ppd-D1* is the major factor affecting flowering time in this germplasm set, explaining 58% of the genotypic variance. Copy number variation at the *Ppd-B1* locus was present but explains only 3.2% and thus a comparably small proportion of genotypic variance. By contrast, the plant height loci *Rht-B1* and *Rht-D1* had no effect on flowering time. The genome-wide scan identified six QTL which each explain only a small proportion of genotypic variance and in addition we identified a number of epistatic QTL, also with small effects. Taken together, our results show that flowering time in European winter bread wheat cultivars is mainly controlled by *Ppd-D1* while the fine tuning to local climatic conditions is achieved through *Ppd-B1* copy number variation and a larger number of QTL with small effects.

## Introduction

Flowering time is one of the most important phenological stages in crop development, as it is key to adaptation, yield potential and yield stability (Snape et al., 2001; Mühleisen et al., 2013). Wheat (*Triticum aestivum* L.) covers more of the world's surface than any other food crop and has the largest production volume of all staple crops in Europe (FAO, 2013). This worldwide expansion and success were possible because of the adaptability of wheat flowering time to different environmental conditions as facilitated by the vast natural variation provided by the hexaploid wheat genome (Worland and Snape, 2001). Understanding the genetic control of flowering time may increasingly gain importance as extreme weather conditions will be expected to occur more frequently already in the near future (Beniston et al., 2007) with potentially negative effects on yield. For example, tailoring flowering time of wheat to local climatic conditions facilitates avoiding high temperatures and drought stress during anthesis and grain filling (Bennett et al., 2012; Bentley et al., 2013). The prediction of flowering time thus plays a key role in adaptation breeding as well as to efficiently transfer promising genotypes into regions with different climatic conditions. Furthermore, in order to increase wheat yield potential, an initiative has recently been started to launch hybrid breeding in wheat (Longin et al., 2012; Whitford et al., 2013). Especially for hybrid seed production, the timing of flowering between male and female parental lines has to be synchronized and in addition, the vulnerability during anthesis can result in decreased pollen production (Pickett, 1993; Longin et al., 2013; Langer et al., 2014).

Flowering time in cereals is controlled by three different signaling pathways: the vernalization (*Vrn*), photoperiod (*Ppd*) and earliness *per se* (*Eps*) pathway (for review see Snape et al., 2001; Distelfeld et al., 2009; Kamran et al., 2014). The group of *Vrn* genes regulates the transition from the vegetative to the generative phase in response to temperature (Distelfeld et al., 2009; Allard et al., 2012) and thus determines winter and spring growth habit. These genes play, however, only a minor role for winter wheat flowering time provided that the vernalization requirement is fulfilled (Kamran et al., 2014). Wheat is a photoperiod sensitive crop and thus flowers only after a critical day length has been reached. However, photoperiod insensitivity has been selected by plant breeders for several decades to enhance yield in certain climatic conditions. *Photoperiod* (*Ppd*) loci genetically control the response to photoperiod, with photoperiod insensitive alleles inducing flowering irrespective of day length. *Ppd-1* encodes a pseudo-response regulator (PRR) (Turner et al., 2005) and the *Ppd-1* homeoloci are located on group 2 chromosomes. *Eps* summarizes all other loci that affect flowering time independently of vernalization and photoperiod response (Worland, 1996; Kamran et al., 2014; Zikhali et al., 2014). Furthermore, different studies reported a moderate but significant correlation between heading time and plant height and Wilhelm et al. (2013) reported a significant effect of *Reduced height* (*Rht*)*-B1* on heading suggesting that genes controlling plant height might also affect flowering time.

For a long time single nucleotide polymorphisms (SNPs) and small insertions-deletions (INDELs) were assumed to be the major types of DNA polymorphisms underlying genotypic variation. However, during the last decade, copy number variation (CNV) was found to be abundant in the human genome (Iafrate et al., 2004; Sebat et al., 2004), affecting the human phenotype and often linked to diseases. By contrast, the extent to which CNVs affect genotypic variation in plants is largely unknown. Copy number variation refers to genomic rearrangements of sequences typically larger than 1 kb, resulting in the gain or loss of DNA segments (Zmienko et al., 2014). Notably, in polyploid plants copy number variation refers to the number of copies per haploid genome. CNVs mainly occur in intergenic regions but can also encompass protein-coding genes or sequences containing regulatory elements. Such CNVs changing the number of functional copies or regulatory elements can affect the expression level of genes. The effects of CNVs have remained undetected in classical QTL mapping experiments because the CNVs are generally not detectable by the commonly used marker systems (e.g., SSRs, DArTs, SNPs). A still small but growing number of reports suggest that copy number variations also contribute to the genotypic variation of important traits in plants, including flowering time (Díaz et al., 2012; Zmienko et al., 2014).

QTL for heading and flowering time in wheat have been identified in several linkage mapping and association mapping studies, mostly based on biparental collections or collections of rather diverse germplasm (Hanocq et al., 2004; Griffiths et al., 2009; Reif et al., 2011b; Rousset et al., 2011; Bennett et al., 2012; Le Gouis et al., 2012; Kamran et al., 2014). The aim of this study was therefore, to employ a candidate gene approach and genotyping-by-sequencing to generate high-density marker data to dissect the genetic architecture of flowering time in European winter bread wheat cultivars. In particular, the objectives of our study were to (1) employ high-density genome-wide association mapping to identify main effect QTL for flowering time based on a population of European winter bread wheat with 410 genotypes evaluated in multi-location field trials, (2) assess the frequency of alleles at *Ppd, Vrn*, and *Rht* candidate genes as well as copy number variation at the *Ppd-B1* locus and to evaluate their effects on flowering time, (3) assess the contribution of epistasis to the genetic architecture of flowering time, and (4) to draw conclusions for plant breeding.

## Materials and methods

### Plant materials, field experiments and meteorological data

A total of 410 winter bread wheat (*T. aestivum* L.) lines were used for this study. Genotypes were European varieties released during the past decades mainly in Austria, Czech Republic, Denmark, Eastern Europe, France, Germany, Poland, Russia, Turkey, and the United Kingdom. The genotypes are referred to as elite germplasm to distinguish it from genotypes not derived from breeding programs. Experiments were conducted in 2012 at three locations in partially replicated designs with a replication rate of 1.27 per location (Williams et al., 2011). Locations were Hohenheim (48°42′50″N, 9°12′58″E, 400 m above sea level (asl), growing season mean temperature 9.6°C and mean precipitation 790 mm, soil type silty loam), Ihinger Hof (48°44′50″N, 8°55′18″E, 493 m asl, growing season mean temperature 8.7°C and mean precipitation 923 mm, soil type silty clay) and Oberer Lindenhof (48°28′26″N, 9°18′12″E, 700 m asl, growing season mean temperature 7.4°C and mean precipitation 1115 mm, soil type silty loam; later referred to as Lindenhof). Entries were sown in observation plots of two rows and 1.25 m length.

Meteorological data were obtained from weather stations directly located at the fields where the experiments were grown (Figure S1A). Hourly air temperature values, measured at 2 m height, were available for Hohenheim and Ihinger Hof and temperatures at 01.00, 07.00, 13.00, and 19.00 h for the station Lindenhof. In order to demonstrate vernalization conditions for each location, cumulative vernalized day degrees were calculated for days from germination employing the method described by Weir et al. (1984) using, however, all available temperature values. Vernalization is best obtained between 3 and 10°C while temperatures between −4 and 3°C as well as between 10 and 17°C result in slower vernalization (Weir et al., 1984; Eagles et al., 2010). For winter wheat, the vernalization requirement can be assumed to be fulfilled when the sum of the accumulated vernalized day degrees has reached 33 vernal days (Weir et al., 1984; Eagles et al., 2010). Days of full vernalization and sum of accumulated vernalized day degrees at January 1st for each location are given in Figure S1B.

Heading date, representing flowering time, was recorded as days after January 1st when 75% of the spikes of an observation plot had emerged to 75% from the flag leaf sheath. Thermal time, often used to more consistently describe the phenological development of plants (Eagles et al., 2010; Rousset et al., 2011; Allard et al., 2012; Cane et al., 2013), was calculated for this time period as the sum of accumulated degree days (°Cd) which are a function of the daily mean temperature and the base temperature of 0°C (Weir et al., 1984).

### Molecular data analysis and candidate genes

All lines were genotyped by genotyping-by-sequencing (GBS) at Diversity Arrays Technology (Yarralumla, Australia) using the Wheat GBS 1.0 assay. Markers with more than 25% missing values and those with a minor allele frequency smaller 0.05 were removed resulting in a total of 23,371 markers for which a map position was available and that were used for the analyses. Associations among the 410 genotypes were analyzed by applying principal coordinate analysis (Gower, 1966) based on the Rogers' distances of the individuals (Wright, 1978) and was done with the software package Plabsoft (Maurer et al., 2008).

For the candidate gene approach, all lines were genotyped for alleles of *Ppd-A1, Ppd-B1, Ppd-D1, Rht-B1, Rht-D1, Rht8-D2, Vrn-A1, Vrn-B1, Vrn-B3*, and *Vrn-D1* (Liu et al., 2012b; Kamran et al., 2014). For the detection of the photoperiod insensitive alleles *Ppd-A1a.1* and *Ppd-B1a.1* (1085bp deletion and 308bp insertion, respectively) and photoperiod sensitive *Ppd-A1b.1* and *Ppd-B1b.1* the approach described by Nishida et al. (2013) was used. Copy numbers for *Ppd-B1* (*Ppd-B1a, Ppd-B1b, Ppd-B1c, Ppd-B1d*, and *Ppd-B1e* indicating 3, 1, 4, 2, and 0 copies, respectively) were detected following the protocol described by Díaz et al. (2012) using, however, [6FAM-BHQ1] and [CY5-BHQ3] labeled probes for *Ppd-B1* and *TaCO2*, respectively, and the Roche LightCycler® 480 System in combination with the Roche LightCycler® 480 Probes Master mastermix. The photoperiod insensitive allele *Ppd-D1a* (candidate causal deletion of 2089bp upstream coding region, ‘Ciano67’ type) and photoperiod sensitive *Ppd-D1b* were detected following the method described by Beales et al. (2007). Allelic variation of *Rht8* was detected employing the diagnostic microsatellite marker WMS 261 (Korzun et al., 1998). Protocols for reduced height alleles causing semi-dwarfism (*Rht-B1b, Rht-D1b*) and tall alleles (*Rht-B1a, Rht-D1a*) were reported by Ellis et al. (2002). Protocols for alleles at *Vrn-A1* (*Vrn-A1a, Vrn-A1b, Vrn-A1c*), *Vrn-B1* (*Vrn-B1, vrn-B1*), *Vrn-B3* (*Vrn-B3, vrn-B3*), and *Vrn-D1* (*Vrn-D1, vrn-D1*) were described by Yan et al. (2004), Chu et al. (2011), Yan et al. (2006), and Fu et al. (2005), respectively.

### Phenotypic data analysis

The phenotypic data were analyzed based on the following statistical model: *y*_ijko_ = μ + *g_i_* + *l_j_* + *gl_ij_* + *r_jk_* + *b_jko_* + *e_ijko_*, where *y_ijko_* was the phenotypic observation of the *i*th wheat line at the *j*th location in the *o*th incomplete block of the *k*th replication, μ was an intercept term, *g_i_* the genetic effect of the *i*th genotype, *l_j_* the effect of the *j*th location, *gl_ij_* the genotype-by-location interaction, *r_jk_* the effect of the *k*th replication at the *j*th location, *b_jko_* the effect of the *o*th incomplete block of the *k*th replication at the *j*th location, and *e_ijko_* was the residual. Error variances were assumed to be heterogeneous among locations. Variance components were determined by the restricted maximum likelihood (REML) method assuming a random model. Significance of variance component estimates was tested by model comparison with likelihood ratio tests. Best linear unbiased estimates (BLUEs) were estimated across locations assuming fixed effects for the genotype. Heritability (*h*^2^) on an entry-mean basis was calculated as the ratio of genotypic to phenotypic variance according to Melchinger et al. (1998). All statistical analyses were performed using ASReml 3.0 (Gilmour et al., 2009).

### Association mapping

For association mapping an additive genetic model was chosen and mapping was done with a mixed model incorporating a kinship matrix as described previously (Yu et al., 2005; Würschum and Kraft, 2014). In brief, the model was: *y_ijp_* = μ + *a_p_* + *g_i_* + *l_j_* + *e_ijp_*, where *y_ijp_* is the adjusted entry mean of the *i*th wheat line at the *j*th location carrying allele *p*, μ the intercept term, *a_p_* the allele substitution effect of allele *p, g_i_* the genetic effect of the *i*th wheat line, *l_j_* the effect of the *j*th location, and *e_ijp_* the residual including the genotype-by-location interaction effect. The allele substitution effect *a_p_* was modeled as fixed effect whereas *g_i_* and *l_j_* were regarded as random effects. The variance of the random genetic effect was assumed to be Var(*g*) = 2Kσ^2^_*G*_, where σ^2^_*G*_ refers to the genetic variance estimated by REML and K was a 410 × 410 matrix of kinship coefficients that define the degree of genetic covariance between all pairs of entries. We followed the suggestion of Bernardo (1993) and calculated the kinship coefficient K_*ij*_ between inbreds *i* and *j* on the basis of marker data as described by Würschum et al. (2011, 2012). For the detection of main effect QTL, a genome-wide scan for marker-trait associations was conducted. To control for multiple testing, we followed the suggestion of Kraakman et al. (2004) and tested at a false discovery rate (FDR) of 0.20 (Benjamini and Hochberg, 1995). The two-dimensional epistasis scan was done based on 2594 equally spaced markers by extending the above model to marker-marker interactions including the subordinated main effects. For the significance level for the epistatic QTL we used an α-level of 0.01 and followed the suggestion of Holland et al. (2002) dividing the α-level by the number of possible independent pairwise interactions between chromosome regions, assuming two separate regions per chromosome (*P* < 1.2e-5). The circular plots illustrating the epistatic interactions were created with Circos (Krzywinski et al., 2009).

The total proportion of genotypic variance (*p_G_*) explained by the detected QTL was calculated by fitting all QTL and the segregating candidate genes simultaneously in a linear model to obtain the adjusted *R*^2^ *R*^2^_*adj*_ which corrects for the number of parameters in the linear model. The ratio *p_G_* = *R*^2^_*adj*_ / *h*^2^, where *h*^2^ refers to the heritability of the trait, yielded the proportion of genotypic variance (Utz et al., 2000). The *p_G_* values of individual QTL were accordingly derived from the sums of squares of the QTL (*SS_QTL_*) in this linear model.

## Results

**Figure 1:**
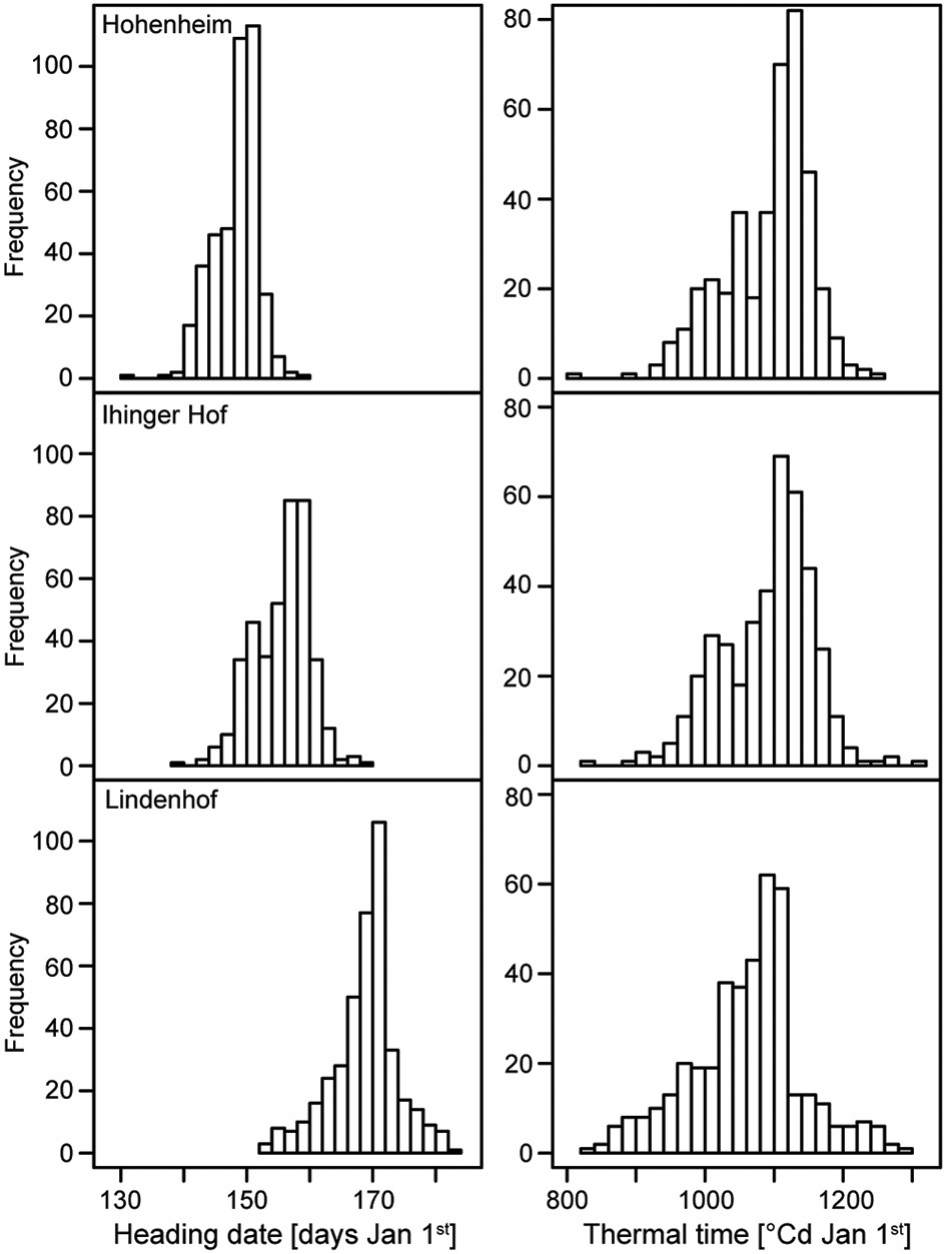
**Histograms of the distribution of the phenotypic values of heading date and thermal time to heading at three locations**.

The 410 winter bread wheat genotypes were evaluated at three locations where the vernalization saturation for winter wheat was reached after 38, 39, and 44 days from germination (Figures S1A,B). The sum of accumulated vernalized day degrees at January 1^st^ was 53.5, 47.0, and 53.9 (Figure S1B). We recorded heading date as days after January 1st and also calculated thermal time to heading by taking into account the temperature per location. The genotypic variance as well as the genotype-by-location interaction variance were significantly larger than zero (*P* < 0.01) for both traits. The heritability estimates were high with 0.93 for heading date and 0.94 for thermal time to heading (Table 1). Heading date BLUEs across locations showed a wide range of 27 days between the earliest and latest variety. The BLUEs per location revealed a strong effect of the location on heading date with Hohenheim being the earliest location and Lindenhof the latest (Figure 1). While the temperature profiles at the three locations ran in parallel, they were highest for Hohenheim and lowest for Lindenhof (Figure S1A). The BLUEs per location for thermal time to heading, i.e., taking the different temperatures at each location into account, largely eliminated the differences between the three locations (Figure 1).

**Table 1:** **Summary statistics for heading date (HD) and thermal time (TT)**.

**Parameter**	**HD**	**TT**
Min	140.60	810.20
Mean	157.60	1082.00
Max	167.70	1238.00
σ^2^_*G*_	13.79^**^	3909.60^**^
σ^2^_*G* × *L*_	0.61^**^	83.10^**^
σ^2^_*e*_	3.35	804.39
*h*^2^	0.93	0.94

Genotypic variance (σ^2^_G_), genotype-by-location interaction variance (σ^2^_G × L_), error variance (σ^2^_e_), and heritability (h^2^).

** *significant at the 0.01 probability level*.

**Figure 2:**
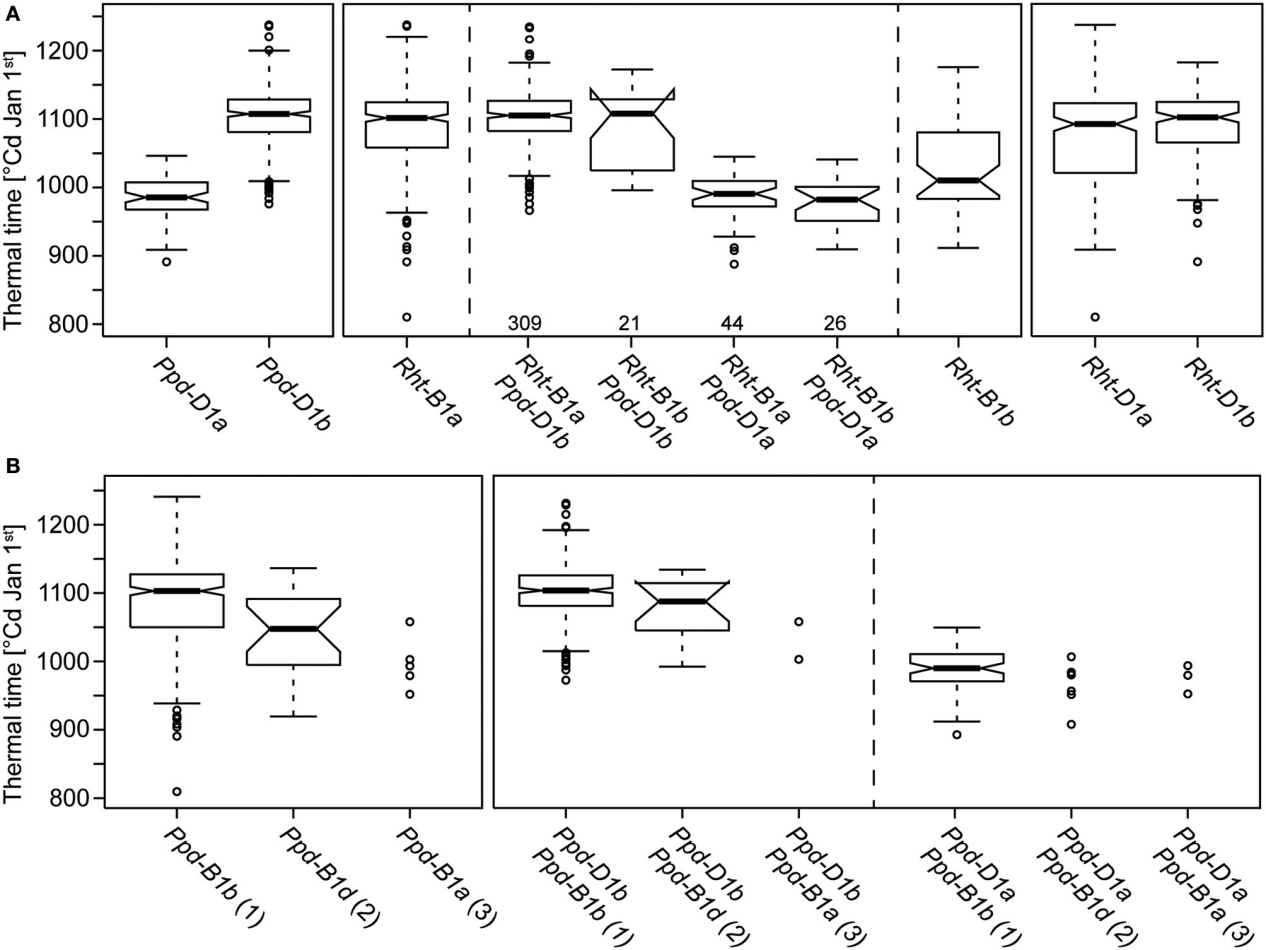
**Boxplots showing thermal time to heading for genotypes carrying different candidate gene alleles. (A)** *Ppd-D1*: *a* (photoperiod insensitive, Ciano67 type), *b* (photoperiod sensitive); *Rht-B1*: *a* (tall allele), *b* (semi dwarfism); *Rht-D1*: *a* (tall allele), *b* (semi dwarfism). Boxplots between the dashed lines represent groups of genotypes with combinations of *Ppd-D1* and *Rht-B1* alleles; numbers underneath refer to the number of genotypes per group. **(B)** *Ppd-B1* copy number alleles identified in this study, *Ppd-B1a, Ppd-B1b* and *Ppd-B1d* (numbers in parentheses indicate the copy number); groups of genotypes with combinations of *Ppd-B1* and *Ppd-D1* alleles.

The candidate gene approach revealed no differences between the 410 genotypes for the *Ppd-A1, Ppd-B1, Vrn-A1, Vrn-B1, Vrn-B3*, and *Vrn-D1* genes. All varieties carried the alleles *Ppd-A1b.1, Ppd-B1b.1, Vrn-A1c, vrn-B1, vrn-B3*, and *vrn-D1*. By contrast, polymorphisms were detected for *Ppd-B1* copy number, *Ppd-D1, Rht8, Rht-B1*, and *Rht-D1*. For the major photoperiod regulator *Ppd-D1*, 18% of the genotypes carried the photoperiod insensitive *Ppd-D1a* allele and 82% the photoperiod sensitive *Ppd-D1b* allele. For the plant height regulator *Rht-B1*, 88% carried the *Rht-B1a* allele and 12% the semi-dwarfing *Rht-B1b* allele, whereas for *Rht-D1* 61% carried the *Rht-D1a* allele and 39% the semi-dwarfing *Rht-D1b* allele. Copy number variation at the *Ppd-B1* locus was also present in this panel of cultivars and the majority of the genotypes had one copy (382), 21 had two copies and 5 had three copies of *Ppd-B1*. Consistent with Cane et al. (2013) we also observed one genotype (‘Naridana’) for which the *Ppd-B1* gene appears to be absent. Genotypes carrying the photoperiod insensitive *Ppd-D1a* allele were substantially earlier flowering than the genotypes with the photoperiod sensitive *Ppd-D1b* allele (Figure 2A). *Ppd-D1* consequently also explained 58.2% of the genotypic variance for thermal time to heading (Table 2). For *Ppd-B1* copy number variation, the genotypes carrying two copies of the gene flowered earlier that the ones with only one copy while the few genotypes with three copies appear to be even earlier (Figure 2B). Increasing copy number at *Ppd-B1* resulted in earlier flowering in a *Ppd-D1b* background while it may have no or only a small effect in plants homozygous for *Ppd-D1a*. While there was no difference in thermal time to heading for the two alleles at the *Rht-D1* locus, we found that the plants carrying the semi-dwarfing *Rht-B1b* allele flowered earlier than the tall *Rht-B1a* plants (Figure 2A). This however, was due to different frequencies of *Ppd-D1a* alleles in both groups. Within plants carrying the *Rht-B1a* allele only 12.5% carried the photoperiod insensitive *Ppd-D1a* allele whereas within the plants carrying the *Rht-B1b* allele 55.3% carried *Ppd-D1a*. After a grouping of the plants according to their allele status at *Ppd-D1*, no differences in thermal time to heading were observed any more between the two *Rht-B1* alleles. Consistently, both *Rht-B1* and *Rht-D1* did not explain any genotypic variation of thermal time to heading (Table 2). By contrast, the height-reducing allele at the *Rht8* locus (192bp allele) reduced thermal time to heading and may even have a small effect in a *Ppd-D1a* background (Figure S4). However, overall the effect of *Rht8* was found to be small as it explained only 1.5% of the genotypic variance (Table 2).

**Table 2:** **Candidate genes and QTL detected for thermal time to heading**.

**Marker/CloneID**	**Chr**.	**Pos**.	***p_G_***	**α-effect**	**Allele frequencies separated for *Ppd-D1* alleles**	
					***Ppd-D1a***	***Ppd-D1b***
**CANDIDATE GENES**						
*Ppd-D1*	2D		58.2	−36.1	0.18	0.82
*Ppd-B1* CNV	2B		3.2	−21.4		
*Rht-B1*	4B		0.0	−3.1	0.07	0.05
*Rht-D1*	4D		0.1	−7.1	0.04	0.36
*Rht8-192bp*	2D		1.5	−4.6	0.09	0.03
**GENOME-WIDE SCAN**						
1089381	5B	12.4	2.0	9.9	0.07	0.70
1006120	5B	104.3	2.2	9.1	0.07	0.27
2297164	5B	209.8	2.9	−22.1	0.12	0.13
1218093	1D	48.3	2.3	11.8	0.08	0.04
994134	6D	184.9	0.1	−1.2	0.18	0.67
**GENOME-WIDE SCAN WITH *Ppd-D1* AS COFACTOR**						
1083683	4B	145.9	1.5	−13.4	0.05	0.09

*Chromosome, position (cM), proportion of genotypic variance explained by the QTL (pG in %), allele substitution (α) effect, and allele frequencies of markers separated according to the Ppd-D1 genotype. QTL allele frequencies refer to the alleles conferring earlier flowering. Rht-B1 and Rht-D1 allele frequencies refer to the dwarfing alleles Rht-B1b and Rht-D1b, respectively*.

**Figure 3:**
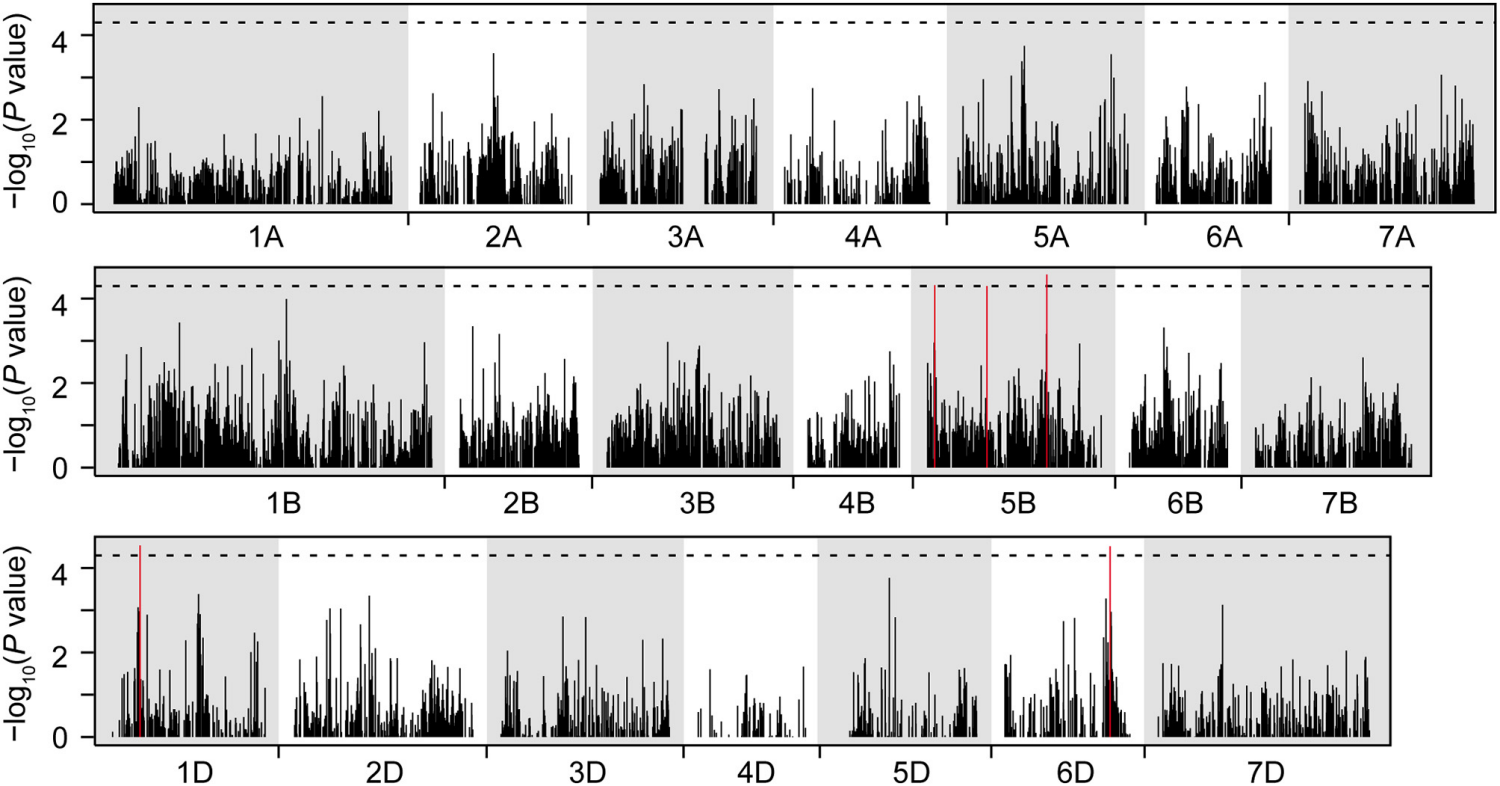
**Genome-wide scan for markers associated with thermal time to heading**. The dashed horizontal line indicates the significance threshold.

**Figure 4:**
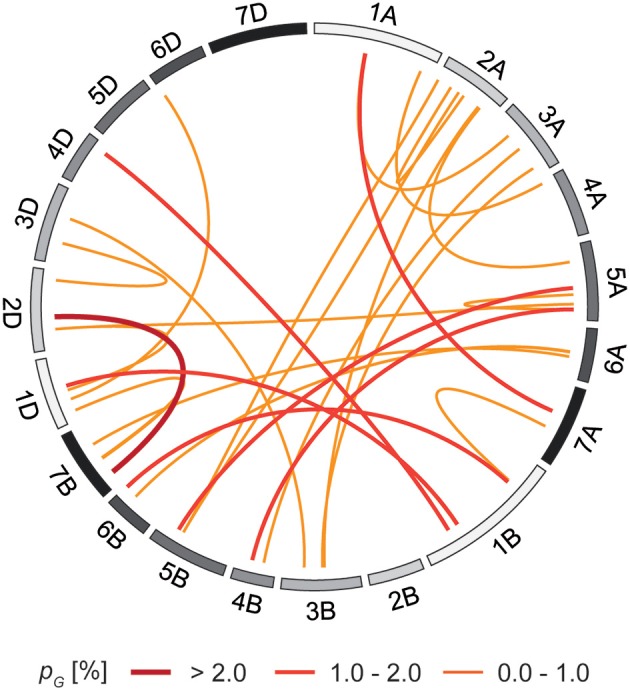
**Epistatic QTL for thermal time to heading**.

The 410 individuals of the mapping population were genotyped by a genotyping-by-sequencing approach which after quality checks yielded 23,371 polymorphic markers with known map position that were used for further analyses. In accordance with previous studies using elite European winter bread wheat (Reif et al., 2011a; Würschum et al., 2013b), the principal coordinate analysis revealed only a slight population structure and the first two principal coordinates explained 19.2 and 8.2% of the variance, respectively (Figure S2). The genome-wide scan for marker-trait associations identified 5 significantly associated markers on chromosomes 5B, 1D, and 6D (Figure 3) which explained between 0.1 and 2.9% of the genotypic variance (Table 2). In addition, we performed a genome-wide scan with *Ppd-D1* as cofactor in the model which revealed another QTL on chromosome 4B explaining 1.5% of the genotypic variance. Together, the five candidate genes and the six identified QTL explained 72.2% of the genotypic variance. The epistasis scan identified 30 significant epistatic interactions (Figure 4) which explained between 0.1 and 2.2% of the genotypic variance (Table S1).

**Figure 5:**
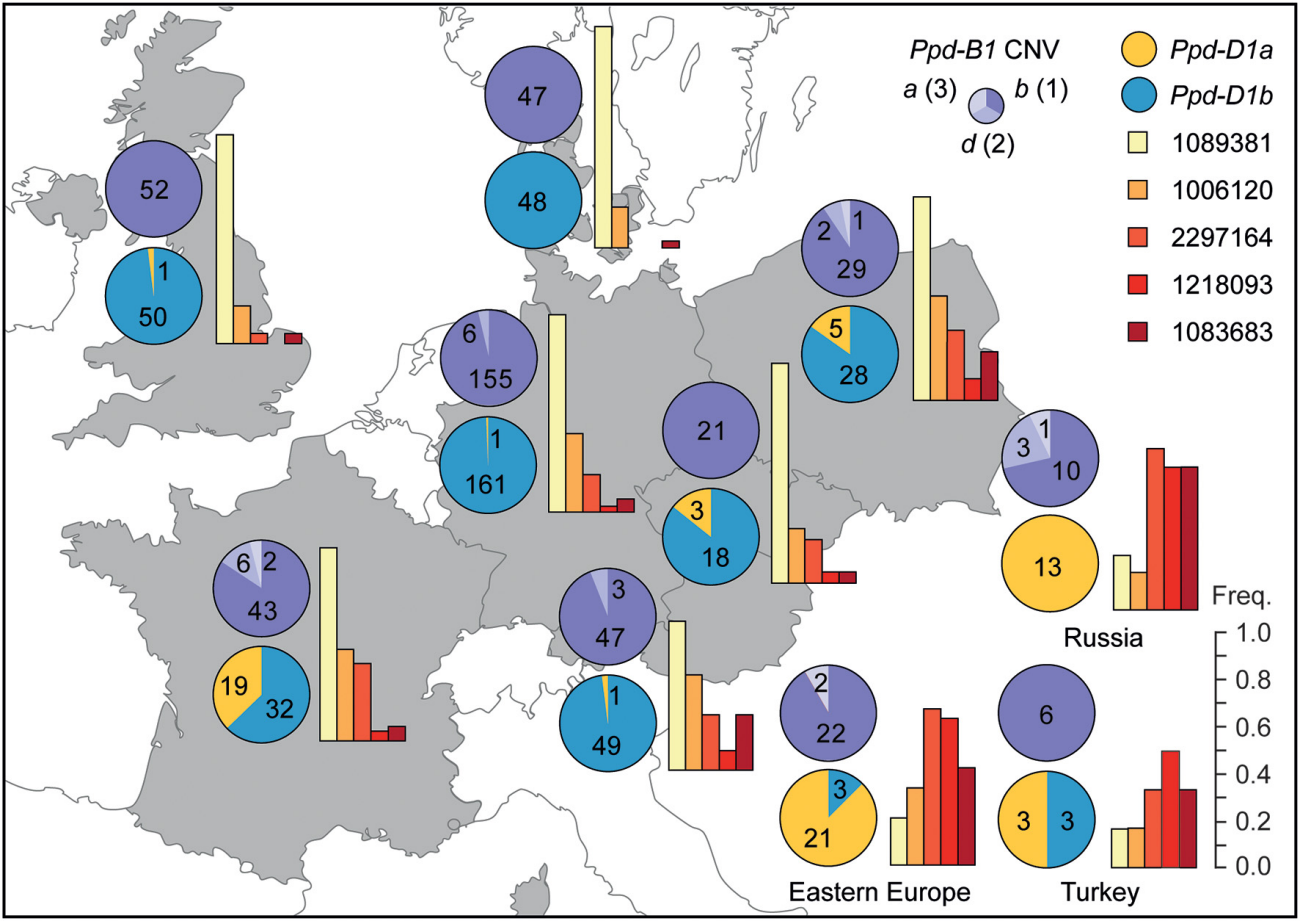
**Distribution of *Ppd-D1, Ppd-B1* copy number and identified QTL alleles in European winter bread wheat in dependence on the country of origin of the variety**. For the QTL the frequency of the allele causing early flowering is shown. Numbers in pie charts indicate the number of varieties for the respective allele. The copy number of the *Ppd-B1a, b*, and *d* alleles is shown in parentheses.

For 379 varieties the country of origin was known and we used this information to analyze the frequency of *Ppd-D1, Ppd-B1* CNV, and QTL alleles dependent on the geographic origin (Figure 5). For *Ppd-D1* the photoperiod insensitive allele *Ppd-D1a* was rare in the UK, Denmark, Germany, Poland, the Czech Republic and in Austria. By contrast, about a third of the French lines carry this allele and in Eastern Europe and Russia it is the predominant allele. For *Ppd-B1* copy number, the one-copy allele is the prevalent allele in all regions and the two-copy allele was mainly found in varieties from the more southern countries. In addition, we analyzed the frequencies of the alleles causing earlier flowering for the detected QTL in the same geographic regions and observed a similar picture. With the exception of one QTL (CloneID 1089381), the allele causing earlier flowering is the minor allele in the first group of countries and occurs at a higher frequency in Eastern Europe and Russia.

## Discussion

### Phenotypic evaluation of flowering time in european winter bread wheat cultivars

Flowering time is of importance in plant breeding as it is central for the adaptation of wheat to different climatic regions and consequently also affects yield potential. The observed genotypic variance of heading date and thermal time to heading was several times larger than the genotype-by-location interaction variance which is in accordance with previous studies in European elite wheat germplasm (Reif et al., 2011b; Langer et al., 2014). All three test locations were located in the south of Germany but we observed strong differences between them with regard to flowering time of the wheat varieties (Figure 1). As vernalization requirement was fulfilled (Figure S1B) and photoperiod was similar for all three locations, temperature remained as a likely cause for the observed differences between the locations which was confirmed by the calculation of thermal time to heading. Taking the temperature at the locations into account eliminated the differences between them observed for heading date. This illustrates the strong effect of temperature on flowering of wheat. However, the low genotype-by-location interaction variance as compared to the genotypic variance indicates that all genotypes responded similarly to the different temperature regimes. Consequently, there appear to be no major QTL for flowering time in response to temperature segregating in European winter bread wheat that affected the trait under the growth conditions represented by our three locations. Nevertheless, a different set of test locations with more diverse temperature profiles may reveal such QTL and the observed genotype-by-location interaction variance may well be caused by medium or small effect temperature response QTL.

The large range in heading date of 28 days (Table 1) can be explained by the different European origins of the varieties included in this study as varieties from southern European countries tended to flower earlier than the more northerly originating ones (Figure S3). Especially in view of the expected climate change, plant breeders need germplasm which allows a flexible response to different climatic conditions. Responding to early summer drought and heat stress, early flowering genotypes can be advantageous provided late frost can be avoided. As earliness is often associated with reduced height and potentially reduced resource capture (Addisu et al., 2009; Bentley et al., 2013) this could be a trade-off regarding yield exploitation. The challenge for future wheat breeding is therefore, to modify flowering time to suit local climatic conditions while maintaining or even increasing yield potential. In order to efficiently exploit the variation and to transfer genotypes between regions, e.g., from southern France to Germany or vice versa, selection tools like marker-assisted selection may be advantageous for future wheat breeding. We, therefore, investigated the genetic control underlying variation of flowering time in the panel of European winter bread wheat varieties by a candidate gene approach and by genome-wide association mapping.

### Candidate genes in european winter bread wheat cultivars and their effect on thermal time to heading

A number of genes involved in the different pathways affecting flowering time were identified so far in wheat and their effects were studied by candidate gene approaches (Eagles et al., 2009, 2010; Rousset et al., 2011; Bentley et al., 2013). In this study we assessed the frequency of some of the known flowering time genes and their contribution to the genetic architecture of thermal time to heading in the panel of European winter bread wheat varieties.

The common spring allele *Vrn-A1a* (Yan et al., 2004) was not detected and the whole panel did not segregate for either of the tested vernalization loci (*vrn-B1, vrn-B3* and *vrn-D1*). This strict winter habit and thus a consequent vernalization requirement is not surprising, as only winter wheat varieties were included in this study. Genes inducing reduced plant height, predominantly the gibberellic acid insensitive alleles of *Rht-B1* and *Rht-D1*, were important components of the “green revolution” (Hedden, 2003). Plant height and its possible effect on flowering time is often discussed as reported associations between flowering time related traits and plant height vary strongly. Langer et al. (2014) observed no correlation in European elite germplasm and neither did Cane et al. (2013) for Australian wheat while other studies did find significant correlations between the two traits (Bordes et al., 2008; Longin et al., 2013; Wilhelm et al., 2013). Furthermore, Wilhelm et al. (2013) reported a significant effect of *Rht-B1* on days to heading. In our study we also observed a significant correlation between plant height and heading time as the short genotypes tended to flower earlier than the tall ones. This effect was mainly due to the *Rht-B1b* allele (Figure 3) and likewise *Rht8* was found to explain a small proportion of the genotypic variance of thermal time to heading (Table 2). This suggested a significant effect of *Rht-B1* on heading date in wheat. However, a more detailed analysis revealed that the differences in thermal time to heading between plants carrying either of the two *Rht-B1* alleles was due to different frequencies of the photoperiod insensitive *Ppd-D1a* allele in these two groups. Within either of the two *Ppd-D1* alleles, *Rht-B1* had no effect on thermal time to heading. Consistently, both *Rht-B1* and *Rht-D1* did not contribute to the genotypic variance when the effect of *Ppd-D1* was taken into account (Table 2). This indicates that the observed correlations between flowering time and plant height are due to different frequencies of *Ppd-D1a* in genotypes with different *Rht* alleles but not to a direct effect of the *Rht* loci on flowering time.

Photoperiodism in wheat is mainly controlled by the *Ppd-D1* locus located on chromosome 2D which greatly influences flowering time by the separation of genotypes into photoperiod sensitive and insensitive ones and accelerates flowering by several days in European environments (Worland, 1996; Beales et al., 2007; Kamran et al., 2014). The major photoperiod insensitive *Ppd-D1a* allele has a 2089bp deletion upstream the coding region which increases expression of the gene and is associated with upregulation of the floral activator *TaFT1* (Beales et al., 2007; Shaw et al., 2012; Bentley et al., 2013). The photoperiod insensitive *Ppd-D1a* allele was introduced into European material in the early Twentieth century when Italian breeders used Japanese germplasm as source (Worland, 1996). Nishida et al. (2013) recently identified alleles of the *Ppd-A1* and *Ppd-B1* loci which accelerate heading in Japanese germplasm and Bentley et al. (2013) showed that photoperiod insensitive alleles at *Ppd-A1* or *Ppd-B1* can have effects as strong as that of *Ppd-D1* when introgressed into hexaploid wheat. However, unlike *Ppd-D1a* neither of these alleles appears to be present in the current European winter wheat material. By contrast, *Ppd-D1a* is present in European winter wheat varieties and genotypes carrying this allele showed a strongly decreased thermal time to heading (Figure 2A). Consistently, this locus also explained the by far largest proportion of genotypic variance (Table 2). This illustrates that not only in biparental populations but also in our panel of European winter wheat varieties *Ppd-D1* is the major determinant of flowering time under fully vernalized conditions. The *Ppd-D1a* allele is mainly present in Eastern and Southern European and Eurasian varieties (Figure 5), as up to the beginning of the twenty-first century photoperiod insensitivity has been introduced into most wheat cultivars grown below 48° latitude (Rajaram and van Ginkel, 2001). The main reason for this is that photoperiod insensitivity is beneficial for crops grown in regions with high summer temperatures in order to avoid heat or drought stress during premature developmental stages (Bentley et al., 2011). In addition, earlier flowering increases yield especially in Southern Europe. The prevalence within Eastern European and Russian varieties may be due to the incorporation of Italian material as reduced photoperiodic sensitivity is advantageous for winter wheat plants adapted to dry land environments, prevailing in the continental climate regions of Russia and the former Soviet Union (Worland et al., 1994; Litvinenko et al., 2001). By contrast, the low proportion of *Ppd-D1a* alleles in varieties originating from northern parts of Europe is due to the considerably lower yield potential of photoperiod insensitive as compared to sensitive varieties in these regions, owing to the shortened vegetative phase.

One of the prime examples for copy number variation in plants is flowering time in wheat which has recently been shown to be affected by CNVs (Díaz et al., 2012). Díaz et al. (2012) showed that *Ppd1* on the B genome (*Ppd-B1*) can be present in different copy numbers. Wheat genotypes with only one copy are photoperiod sensitive whereas an increased copy number (2–4 copies) results in a day-neutral, early flowering phenotype. These experiments were conducted on a rather limited number of lines and with phenotypic data from controlled greenhouse conditions. Cane et al. (2013) found all four alleles to be present in southern Australian wheat and reported that the three-copy allele (termed *Ppd-B1a*) and the four-copy allele (*Ppd-B1c*) reduced days to heading as compared to the one-copy allele (*Ppd-B1b*) whereas the two-copy allele (*Ppd-B1d*) increased days to heading. In our collection of European winter wheats, the majority of the cultivars carried the one-copy allele and only few the two- or three copy alleles. The allele with four copies which is characteristic for Chinese spring was not present in our collection, in contrast to Cane et al. (2013) who found this allele in a number of modern Australian cultivars and current breeding lines. In contrast to the findings of Cane et al. (2013), our results indicate that both the two- and the three-copy alleles reduce the time to heading in our panel and under the growth conditions present at our test locations (Figure 2B). The proportion of genotypic variance explained by *Ppd-B1* copy number variation under field conditions was small compared with *Ppd-D1*, however still larger than that of any of the detected QTL (Table 2).

Taken together, our candidate gene analyses revealed that the *Reduced height* loci appear to have no effect on flowering time in European winter bread wheat varieties and confirmed *Photoperiod-D1* as the major source of variation of flowering time. In addition to the major effect of *Ppd-D1*, copy number variation at the *Ppd-B1* locus may add in the fine-tuning of adaptation of European winter wheat to local climatic conditions.

### QTL and epistatic QTL for thermal time to heading

Even though *Ppd-D1* explained 58% of the genotypic variance, there is still variation for flowering time in European winter bread wheat not explained by this major regulator. In order to identify additional components of the genetic architecture underlying the trait we performed a genome-wide association mapping in our panel of varieties. This identified six small effect QTL (Figure 3, Table 2) indicating that beside *Ppd-D1* there are no other major QTL affecting flowering time in European winter bread wheat. Rather, the fine tuning to local climatic conditions appears to be controlled by many small effect QTL. With regard to a knowledge-based breeding, such small effect QTL that escape detection in QTL mapping approaches could probably be better captured by genome-wide prediction approaches which may be an option for the future and warrants further research. While it is not possible to unambiguously assign the detected QTL to one of the flowering pathways, the QTL on chromosome 1D (CloneID 1218093) might be part of the *Eps* signaling pathway as Zikhali et al. (2014) recently reported an *Eps* QTL on this chromosome.

The geographic pattern of distribution of alleles of the six QTL resembled the distribution of the two *Ppd-D1* alleles. The allele conferring earlier flowering was more prominent in the varieties originating from southern regions than in the northern regions suggesting that these QTL contribute to the adaptation of winter wheat varieties to different European climatic conditions. The total proportion of genotypic variance explained by *Ppd-D1* and the detected QTL was 72.2% suggesting that there are other QTL with effects too small to be detected or with medium or large effects which remained undetected. Surprisingly, we did not identify a QTL on chromosome 2D in our genome-wide scan, despite *Ppd-D1* being located on this chromosome. We did, however, identify an unmapped marker (CloneID 3940868) that of all tested markers was most strongly associated with the trait and which is located in the promotor region of *Ppd-D1*. The linkage disequilibrium (LD) between this marker and any of the mapped markers on chromosome 2D was low suggesting a rapid decay of LD around this locus in this germplasm. Bentley et al. (2013) have recently attempted to develop markers flanking *Ppd* but found none of them to be polymorphic on their lines suggesting that the region around *Ppd* may be largely monomorphic. Our result illustrates that despite the comparably high number of markers obtained by the genotyping-by-sequencing approach there are still chromosomal regions that are not covered with this marker density and consequently regions where QTL may have remained undetected.

Another potential source for the variance not accounted for by *Ppd-D1* and the detected QTL is epistasis. Epistasis refers to interactions between two or more loci in the genome (Carlborg and Haley, 2004) and has recently been shown to contribute to the genetic architecture of complex traits in different crops including maize, wheat and rapeseed (Buckler et al., 2009; Reif et al., 2011b; Liu et al., 2012a; Steinhoff et al., 2012; Würschum et al., 2013a). Reif et al. (2011b) showed the contribution of epistasis to the genetic architecture of flowering time in elite winter wheat and in addition, Bentley et al. (2013) have recently shown a dependency of the effect of *Ppd-D1a* alleles on the genetic background. Consistent with these previous findings, we also identified epistatic QTL for thermal time to heading. While the proportion of genotypic variance explained by individual epistatic QTL was small, combined they could contribute a substantial proportion to the observed variation for flowering time. In conclusion, the genome-wide scan identified only six QTL suggesting that in addition to the major regulator *Ppd-D1* the genetic architecture of flowering time in European winter bread wheat is controlled by many QTL with only small effects and potentially by epistasis.

## Conclusions

In this study we employed a large panel of European winter bread wheat varieties to unravel the genetic architecture underlying flowering time in this germplasm set. Using a candidate gene approach and genome-wide association mapping, we show that in fully vernalized winter wheat more than half of the genotypic variation is attributable to the major photoperiod regulator *Ppd-D1*. The remaining variation appears to be due to copy number variation at the *Ppd-B1* locus, other small effect QTL and epistatic QTL. With regard to a knowledge-based breeding of wheat these results suggest that only *Ppd-D1* is worth to be included in marker-assisted selection programs whereas the subsequent fine tuning to local conditions is better done based on phenotypic selection in the field.

### Conflict of interest statement

The authors declare that the research was conducted in the absence of any commercial or financial relationships that could be construed as a potential conflict of interest.
