# Supplementary material for: Flowering time control in European winter wheat
Source: Front Plant Sci. 2014 Oct 9;5:537. doi: 10.3389/fpls.2014.00537 (PMC4191279; doi:10.3389/fpls.2014.00537)
Supplement: Supplementary file 1 [file Presentation1.PDF]

Flowering time control in European winter wheat

Simon M. Langer, C. Friedrich H. Longin, Tobias Würschum

Supplementary Material

Table S1 Epistatic QTL detected for thermal time to heading. Chromosome and position (cM) of both interacting markers, proportion of genotypic variance explained by the epistatic QTL (p_G in %), and allele substitution (α) effect.

Marker 1	Marker 1 Chr.	Marker 1 Pos.	Marker 2	Marker 2 Chr.	Marker 2 Pos.	p_G	α-effect
1159421	1A	205.9	1090692	3A	73.8	0.5	-7.4
1212780	1A	209.3	1225056	7A	116.0	0.3	3.7
1126208	1A	445.1	1106383	4A	7.3	0.1	-2.4
1144414	2A	4.1	2297164	5B	209.8	NA	NA
1267706	2A	101.7	2297164	5B	209.8	NA	NA
1122848	2A	63.7	1212547	2A	126.1	0.1	-6.1
1010498	2A	200.0	2358107	3B	136.0	0.6	7.8
1068421	2A	207.7	1228444	5A	59.9	0.1	2.4
1204957	3A	143.4	1119045	4B	49.8	0.4	-6.1
2258878	3A	240.0	2276021	3B	143.8	0.6	6.5
1258755	5A	166.2	990481	5B	232.0	1.5	-14.4
3064895	5A	193.0	2304155	5A	253.0	0.9	9.4
2374022	5A	231.8	2249013	2D	83.4	0.1	-2.0
1144606	1D	128.7	2249013	2D	83.4	0.4	6.1
2304155	5A	253.0	1048383	4D	98.1	1.2	-11.1
1110069	6A	96.1	1126217	6B	116.4	0.3	-4.9
1024924	6A	98.2	1126217	6B	116.4	0.3	5.5
1128442	6A	116.8	1254230	7B	188.8	0.5	-10.1
1122258	7A	184.1	1122909	1B	112.4	1.7	16.7
1122909	1B	112.4	3022152	6B	172.0	0.3	-6.0
1082141	1B	123.9	3022152	6B	172.0	1.9	-10.2
1109266	1B	391.8	2322336	2D	146.9	1.0	-6.1
1125039	1B	388.0	2322336	2D	146.9	1.0	6.3
1253592	1B	431.3	1116213	4D	167.1	1.6	13.7
1126376	3B	218.5	2245455	3D	192.0	0.7	6.4
1069235	7B	51.8	987378	2D	133.6	2.2	13.1
1097823	7B	120.4	1061993	2D	41.3	0.2	-4.4
1156277	7B	124.6	1061993	1D	41.3	0.1	-2.2
3222341	1D	90.1	2281208	6D	6.5	0.4	-5.7
1104828	2D	281.9	1089038	3D	88.6	0.2	4.5

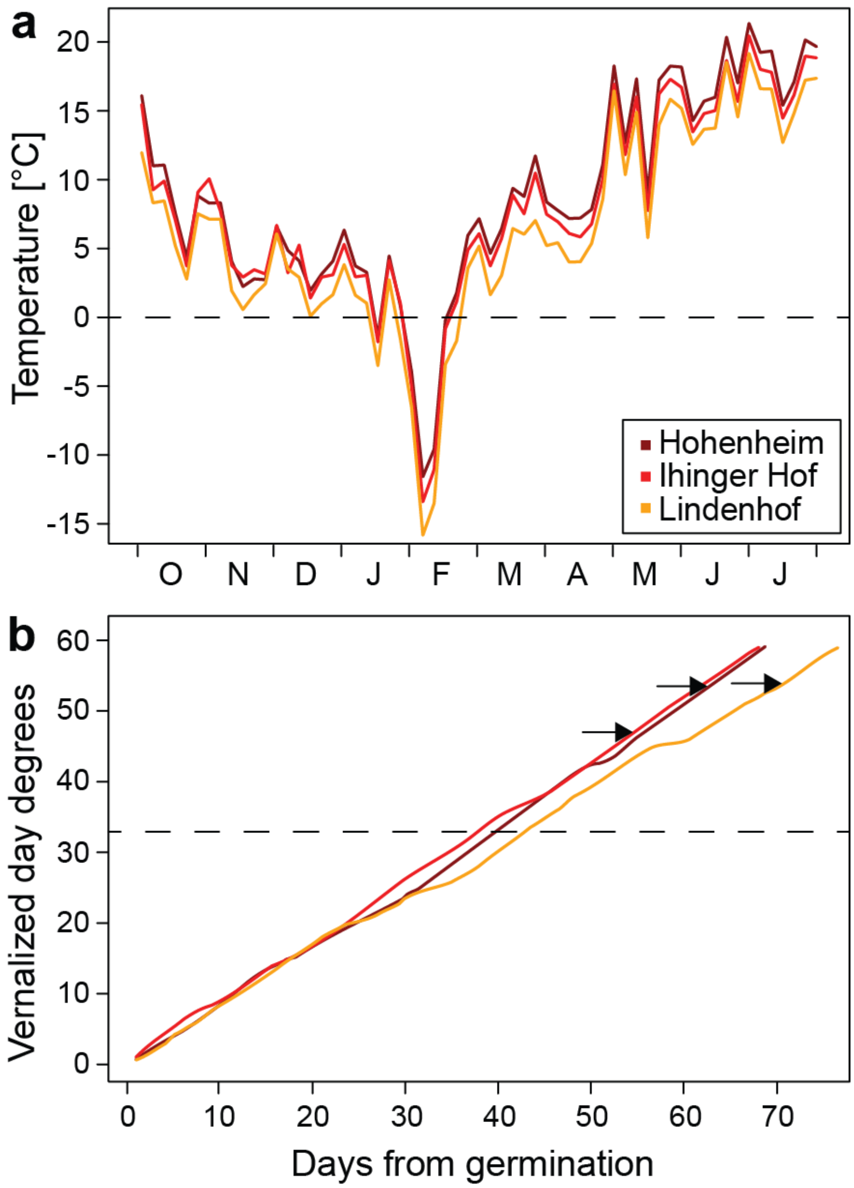

Figure S1 Thermal conditions at the three experimental stations. (**a**) Five days average air temperature (°C) curves for the time period from October to July, the dashed line marks the 0°C level. (**b**) Accumulated vernalized day degrees from the day of germination. The dashed line marks the sum of 33 vernal day units as the full vernalization threshold for winter wheat; arrows mark the accumulated vernalized day degrees at January 1st for each of the three locations, 53.5 for Hohenheim, 47.0 for Ihinger Hof and 53.9 for Lindenhof.

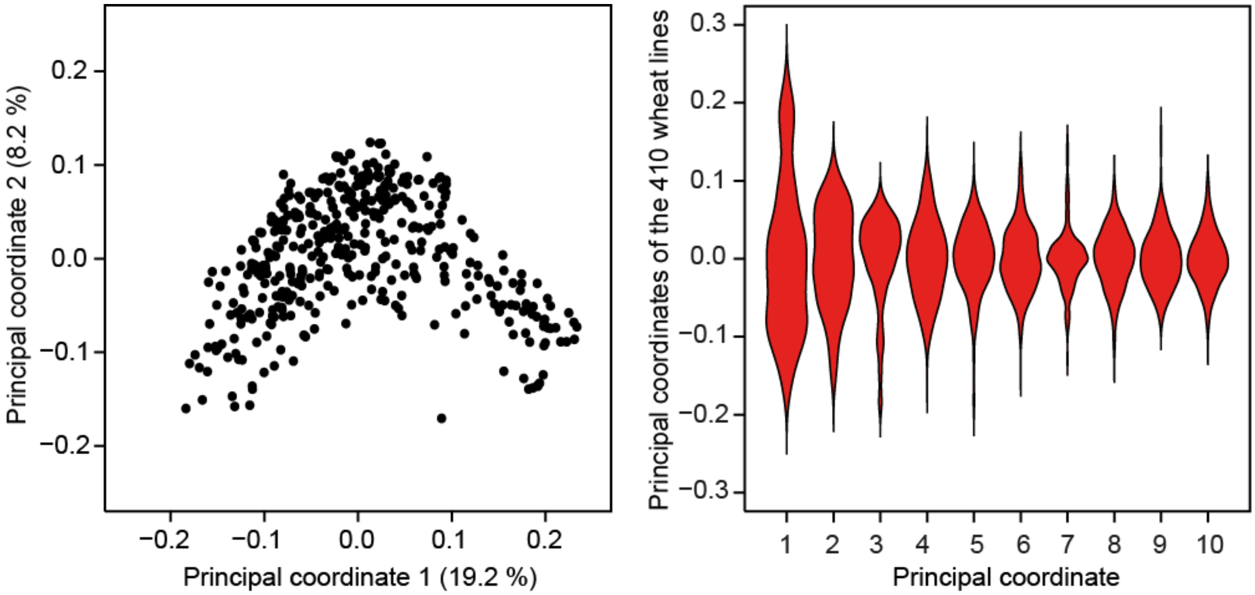

Figure S2 (**a**) Population structure analyzed by principal coordinate analysis of the 410 genotypes based on modified Rogers' distance estimates. Percentages refer to the proportion of variance explained by the principal coordinate. (**b**) Violin plot showing the density distribution of the first ten principal coordinates.

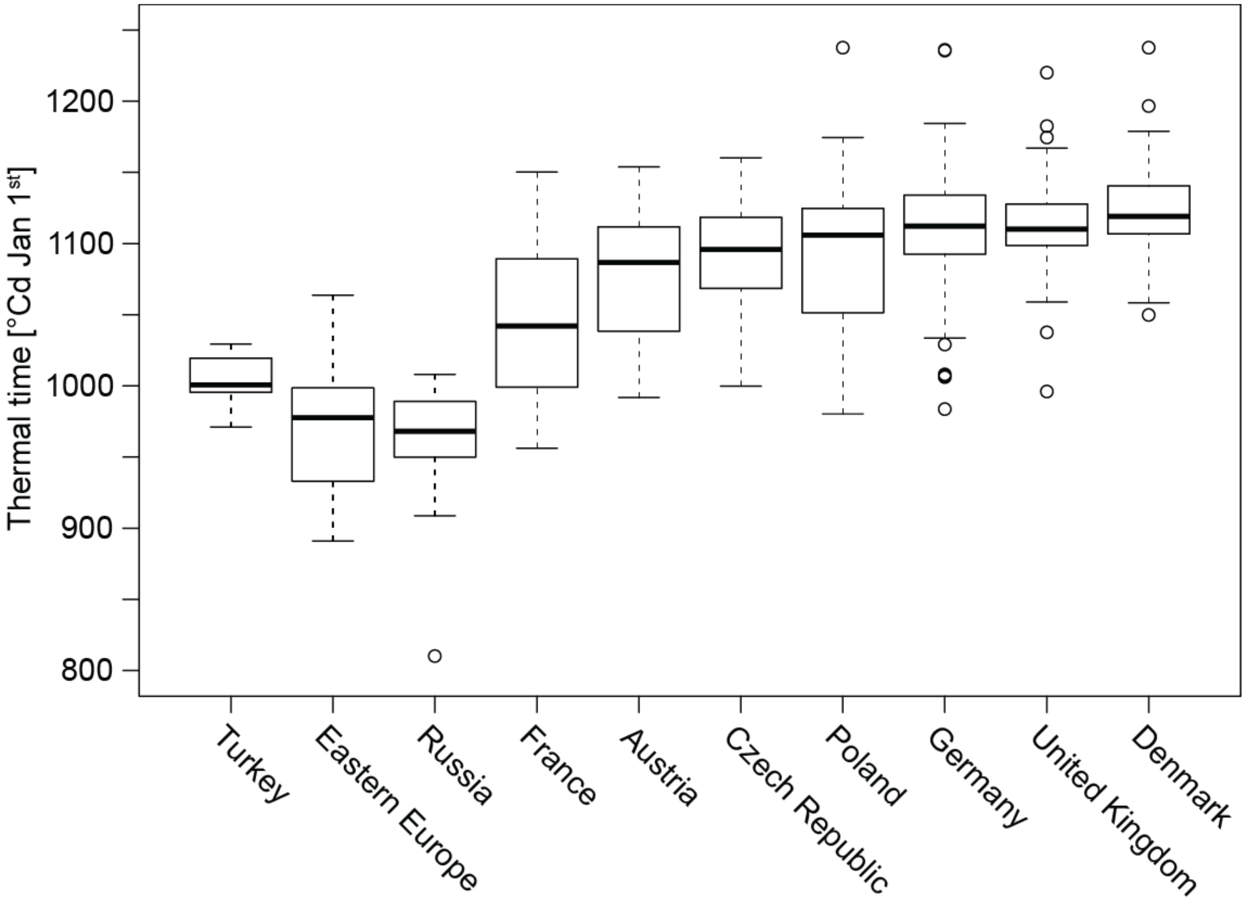

Figure S3 Boxplots showing thermal time to heading for varieties originating from different countries.

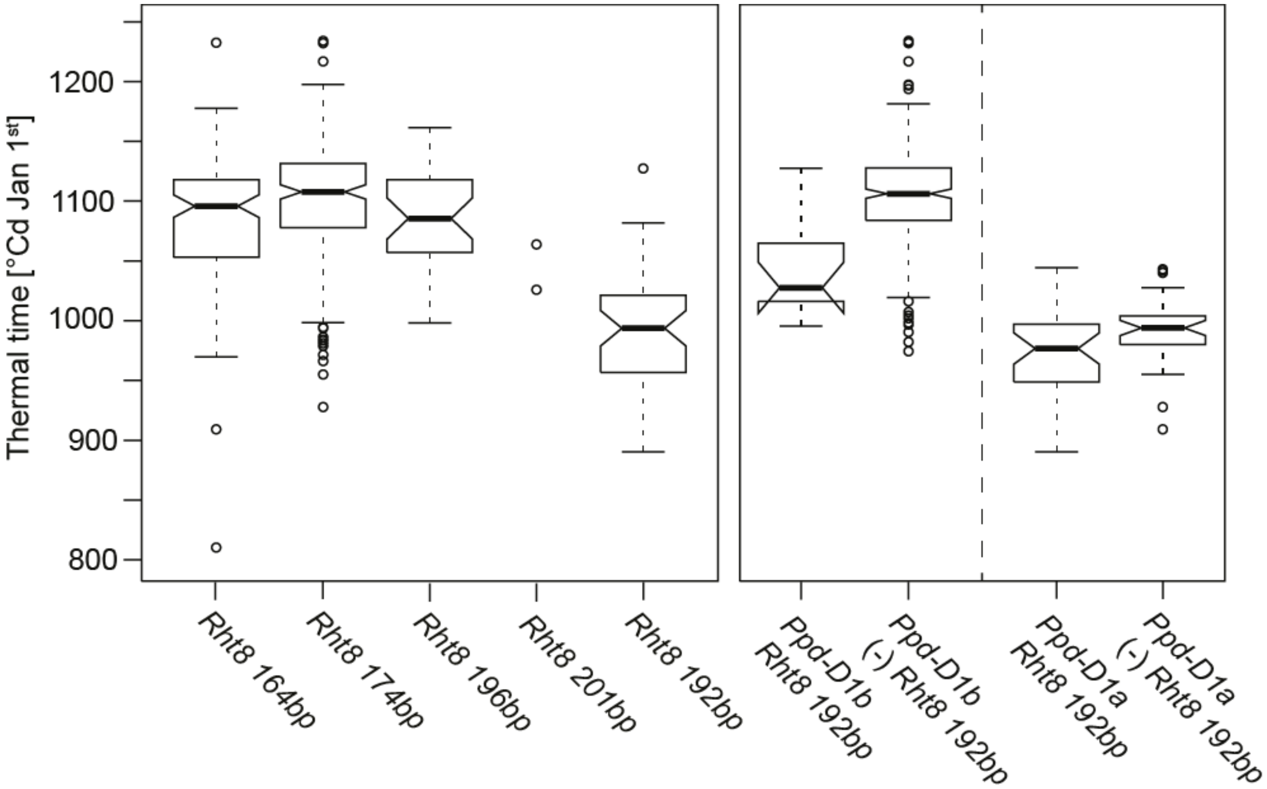

Figure S4 Boxplots showing thermal time to heading for genotypes carrying identified *Rht8* alleles (left box) and groups of genotypes with combinations of *Ppd-D1* and *Rht8 192bp* alleles; '(-)' indicates genotypes without the *Rht8 192bp* allele.
